# Lower serum C3 levels are associated with glomerulosclerosis in membranous nephropathy and ANCA-glomerulonephritis

**DOI:** 10.1093/ckj/sfag350

**Published:** 2026-10-05

**Authors:** Martina Tedesco, Elisa Delbarba, Martina Uzzo, Gianmarco Lugli, Chiara Bedognè, Marta Salti, Francesco Ravelli, Corrado Damilano, Eva Sestagalli, Marta Arrighini, Simona Fisogni, Vincenzo L’Imperio, Fabio Pagni, The REASSESS Study Group, Jennifer Scott, Jennifer O’Brien, Kresimir Galesic, Simonetta Genovesi, Giorgio Trivioli, Marco Allinovi, Iva Gunnarsson, Anna Juto, Robert Hall, Silke R. Brix, Federica Maritati, Anisa Idrizi, Anna Ricchiuto, Annette Bruchfeld, Gaetano La Manna, Mario Cozzolino, Renato A Sinico, Augusto Vaglio, Giacomo Emmi, Ingeborg Bajema, Andreas Kronbichler, Matija Crnogorac, Stephen McAdoo, Mark A Little, David R W Jayne, Federica Mescia, Federico Alberici

**Affiliations:** Department of Clinical and Experimental Sciences, University of Brescia, Brescia, Italy; Nephrology Unit, ASST Spedali Civili di Brescia, Brescia, Italy; Nephrology Unit, ASST Spedali Civili di Brescia, Brescia, Italy; Nephrology and Dialysis Division, IRCCS Humanitas Research Hospital, Milan, Italy; Department of Biomedical Sciences, Humanitas University, Milan, Italy; Department of Medical and Surgical Specialties, Radiological Sciences and Public Health, University of Brescia, Brescia, Italy; Nephrology Unit, ASST Spedali Civili di Brescia, Brescia, Italy; Department of Medical and Surgical Specialties, Radiological Sciences and Public Health, University of Brescia, Brescia, Italy; Nephrology Unit, ASST Spedali Civili di Brescia, Brescia, Italy; Department of Clinical and Experimental Sciences, University of Brescia, Brescia, Italy; Nephrology Unit, ASST Spedali Civili di Brescia, Brescia, Italy; Department of Medical and Surgical Specialties, Radiological Sciences and Public Health, University of Brescia, Brescia, Italy; Nephrology Unit, ASST Spedali Civili di Brescia, Brescia, Italy; Department of Medical and Surgical Specialties, Radiological Sciences and Public Health, University of Brescia, Brescia, Italy; Nephrology Unit, ASST Spedali Civili di Brescia, Brescia, Italy; Department of Medical and Surgical Specialties, Radiological Sciences and Public Health, University of Brescia, Brescia, Italy; Nephrology Unit, ASST Spedali Civili di Brescia, Brescia, Italy; Pathology Unit, ASST Spedali Civili di Brescia, Brescia, Italy; Pathology, Fondazione IRCCS San Gerardo Dei Tintori, Monza, Italy; School of Medicine and Surgery, University of Milano-Bicocca, Milan, Italy; Pathology, Fondazione IRCCS San Gerardo Dei Tintori, Monza, Italy; School of Medicine and Surgery, University of Milano-Bicocca, Milan, Italy; Department of Health, Medicine and Caring Sciences, Linköping University Hospital, Linköping, Sweden; Department of Clinical Science, Intervention and Technology, Division of Renal Medicine, Karolinska Institutet, Stockholm, Sweden; Nephrology Dialysis and Kidney Transplant Unit, IRCCS Azienda Ospedaliero-Universitaria di Bologna, Bologna, Italy; Department of Medical and Surgical Sciences (DIMEC), Alma Mater Studiorum-University of Bologna, Bologna, Italy; Department of Health Sciences, University of Milan, Milan, Italy; Kidney Division, ASST Santi Paolo e Carlo, Milan, Italy; Nephrology and Dialysis Division, IRCCS Humanitas Research Hospital, Milan, Italy; Department of Biomedical Experimental and Clinical Sciences “Mario Serio”, University of Florence, Florence, Italy; Nephrology and Dialysis Unit, Meyer Children’s Hospital IRCCS, Florence, Italy; Centre Hospitalier Universitaire Vaudois Immunology and Allergy Service, University of Lausanne, Lausanne, Switzerland; Department of Medical Surgical and Health Sciences, University of Trieste, Trieste, Italy; Centre for Inflammatory Diseases, Department of Medicine, Monash Medical Centre, Monash University, Melbourne, Victoria, Australia; Department of Pathology and Medical Biology, University Medical Center, University of Groningen, Groningen, Netherlands; Department of Internal Medicine IV (Nephrology and Hypertension), Medical University Innsbruck, Innsbruck, Austria; Department of Nephrology and Dialysis, Dubrava University Hospital, Zagreb, Croatia; Renal & Transplant Centre, Hammersmith Hospital, Imperial College Healthcare NHS Trust, London, UK; Vasculitis Centre, Department of Immunology & Inflammation, Imperial College London, London, UK; Trinity Kidney Centre, Trinity Translational Medicine Institute, School of Medicine, Trinity College Dublin, Dublin, Ireland; Department of Medicine, University of Cambridge, Cambridge, UK; Nephrology Unit, ASST Spedali Civili di Brescia, Brescia, Italy; Department of Medical and Surgical Specialties, Radiological Sciences and Public Health, University of Brescia, Brescia, Italy; Department of Medical and Surgical Specialties, Radiological Sciences and Public Health, University of Brescia, Brescia, Italy; Nephrology Unit, ASST Spedali Civili di Brescia, Brescia, Italy

**Keywords:** ANCA-glomerulonephritis, C3, complement, glomerulosclerosis, membranous nephropathy

## Abstract

**Background:**

Despite the key pathogenetic role of the complement cascade in various immune-mediated kidney diseases, the clinical significance of serum C3/C4 levels in conditions where most patients exhibit complement levels within normal limits, such as membranous nephropathy (MN) and ANCA (antineutrophil cytoplasmic antibody)-glomerulonephritis (ANCA-GN), remains underexplored. This study focused on the clinico-pathological correlates of serum complement C3/C4 levels in two cohorts of patients with biopsy-proven MN and ANCA-GN.

**Methods:**

Based on median C3 and C4 levels at time of diagnostic biopsy, the cohorts were stratified into four groups, referred to as “complement component-high” and “-low.” Clinico-pathological features were compared between these groups and correlated with C3/C4 levels.

**Results:**

In the MN cohort (*n* = 44), patients in the C3-low group showed a higher degree of global (8.3% vs 0%, *p* = .015) and combined global and segmental glomerulosclerosis (9.5% vs 0%, *p* = .032) compared to the C3-high group. In the ANCA-GN cohort (*n* = 198), serum C3 levels were lower in cases with the sclerotic class according to Berden classification (107 [98–123] mg/dl) compared to those classified as focal (138 [112–151] mg/dl, *p* < .001), crescentic (127 [107–151] mg/dl, *p* = .003), or mixed (123 [101–140] mg/dl, *p* = .018). Furthermore, ANCA-GN patients in the C3-low group showed lower estimated glomerular filtration rate at presentation and serum C3 was inversely associated with the risk of kidney failure, independently of several confounders (Hazard ratio [HR] for 10 mg/dl increase in C3: 0.91, 95% Confidence Interval [CI] 0.83–1.00, *p* = .049).

**Conclusions:**

Serum C3 levels in the low-normal range were associated with glomerulosclerosis in both ANCA-GN and MN, and with poorer kidney outcome in ANCA-GN, supporting a potentially cross-disease role of the complement cascade in mediating glomerular injury.

KEY LEARNING POINTS **What was known:**

- Complement plays an important role in several glomerular diseases, including membranous nephropathy (MN) and ANCA-glomerulonephritis (ANCA-GN). While complement activation has been demonstrated during disease activity in both MN and ANCA-GN, it remains unclear whether serum C3/C4 levels bear clinical relevance in the setting of these glomerular diseases.

**This study adds:**

- This study explored the clinico-pathological correlates of serum complement C3 and C4 levels in two independent cohorts of patients with biopsy-proven MN and ANCA-GN. Serum C3 levels in the low–normal range were associated with glomerulosclerosis in both ANCA-GN and MN, and with poorer kidney outcome in ANCA-GN.

**Potential impact:**

- These results suggest a potential cross-disease role of complement activation in glomerular injury, particularly sclerosis. While this has important therapeutic implications, more specific complement biomarkers—beyond serum C3/C4—are needed to better reflect underlying pathophysiology and guide targeted treatment strategies.

## INTRODUCTION

Insights into complement biology revealed involvement of the complement system in several glomerular diseases, including membranous nephropathy (MN) and ANCA (antineutrophil cytoplasmic antibody)-glomerulonephritis (ANCA-GN) [1–3]. Despite not being the primary driver of disease pathogenesis in either condition, complement plays an important role as a secondary mediator of injury in both [4]. In Anca-Associated Vasculitis (AAV), despite the absence of deposition of complement and immunoglobulins in affected tissue (“pauci-immune”) in most cases, complement exerts damage primarily through fluid-phase activation by ANCA-primed neutrophils [3, 5]. It is well established that complement activation in ANCA-GN primarily involves the alternative and terminal pathways [3, 6, 7]. In particular, the C5 axis plays an important role in neutrophils priming, generating a positive inflammatory loop mediated by the alternative pathway, whereby neutrophil degranulation further promotes C5a generation, ultimately leading to endothelial damage [7]. Genetic deletion of C5 [6] or pharmacological blockade of C5a [8] protects against the development of necrotizing crescentic glomerulonephritis in mice; moreover, a growing body of evidence supports complement inhibition in ANCA-GN [9], ranging from completed studies of the C5a receptor antagonist avacopan [10–12] and the C5a inhibitor vilobelimab [13] to ongoing trials evaluating agents targeting the alternative complement pathway (NCT07160608 [14], NCT06388941 [15]).

In primary MN, the activation of all three complement pathways has been described [1, 2]. Of note, C3a/C3aR signaling plays a central role in inducing albumin leakage in *in vitro* and murine models of MN [16]. Furthermore, the insertion of the Membrane Attack Complex (MAC) into the podocyte membrane triggers cytoskeletal disarrangement, inducing the production of reactive oxygen species and, ultimately, cellular apoptosis and detachment [1, 2, 17]. Importantly, increased circulating split products of the complement cascade have been documented during active disease in both AAV and MN [18–20].

While measurement of complement activation biomarkers is poorly standardized and not readily accessible, determination of serum complement C3 and C4 levels is widely available in routine clinical practice. It remains unclear whether serum C3/C4 levels bear clinical relevance in the setting of glomerular diseases where overt hypocomplementemia is a rare occurrence, such as MN and ANCA-GN. The main goal of our study was to explore the clinico-pathological correlates of serum complement C3 and C4 levels in two independent cohorts of patients with MN and ANCA-GN.

## MATERIALS AND METHODS

### Study cohorts

This was a single-center retrospective study for the MN cohort and a multicenter retrospective study for the ANCA-GN cohort. The MN cohort was enrolled at the Nephrology Unit of ASST Spedali Civili of Brescia. Inclusion criteria were: a clinico-histological diagnosis of primary MN between 2010 and 2023, age at diagnosis ≥18 years, and availability of serum complement levels at diagnosis (Fig. S1). A multicenter cohort of patients with biopsy-proven ANCA-GN, collected across 11 centers (Table S1) of the European Vasculitis Society as part of an independent project [21], was also analyzed. For this cohort, the subset of patients with available serum complement levels at diagnosis was included in the study (Fig. S1).

The study was carried out under the ethics of the “Coorte MaRe” project, approved by the Ethics Committee of ASST Spedali Civili di Brescia (ethics number 4945) for the Brescia cohort. For the multicentric AAV cohort, the study was performed in compliance with local ethics requirements.

### Data collection

Demographic characteristics and laboratory data at time of kidney biopsy were collected and compared across complement groups. Estimated glomerular filtration rates (eGFRs) were calculated using the 2009 Chronic Kidney Disease Epidemiology Collaboration (CKD-EPI) equation [22]. In the MN cohort, anti-PLA2R seropositivity was defined based on positive serum assay [indirect immunofluorescence (IIF) or ELISA]. For the ANCA-GN cohort, additional baseline data were collected regarding AAV phenotype, ANCA specificity by ELISA, baseline organ involvement, and disease activity using the Birmingham Vasculitis Activity Score (BVAS) version 3.0 [23]. Key clinical outcomes were recorded in both cohorts, including all-cause mortality and kidney failure (KF), the latter defined as kidney failure with replacement therapy (dialysis or kidney transplant) or the occurrence of CKD stage V in at least two consecutive time-points, including the last follow-up visit. Relevant histological findings were extrapolated from the diagnostic kidney biopsy reports. For the MN cohort, these were generated by two expert pathologists at ASST Spedali Civili di Brescia. For the ANCA-GN cohort, diagnostic biopsy reports were generated by expert renal pathologists at each participating tertiary referral center. No central revision of biopsy specimens was performed. In the MN cohort, the following histological parameters were collected: glomerulosclerosis (GS), either global or segmental, expressed as the percentage of affected glomeruli; interstitial fibrosis and tubular atrophy, categorized as absent, mild, moderate, or severe; mesangial and endocapillary hypercellularity; glomerular staining by IIF for Immunoglobulin G (IgG) and C3 were scored semi-quantitatively from 0 to 4+. In the ANCA-GN cohort, the distribution of Berden classes [24] was assessed.

### Statistical analysis

Patients were stratified into four complement groups according to levels of C3 and C4 at time of kidney biopsy equal/greater than or below the respective median values, referred to as “C3-high”/“C4-high” and “C3-low”/“C4-low,” respectively. Continuous variables were reported as mean ± SD for normally distributed data, and as median with interquartile range (IQR) for non-normally distributed or ordinal data. Categorical variables were expressed as counts and percentages (*n*/*N*, %). Clinical, laboratory, and histological features were compared across complement groups using the unpaired Student’s *t*-test, Mann–Whitney *U* test, or Fisher’s exact test, as appropriate. A two-sided *P*-value <.05 was considered statistically significant. In the MN cohort, the association between serum complement and selected histological parameters was assessed using Spearman’s rank correlation, showing statistically significant correlations in a correlogram. The probability of achieving a complete remission (proteinuria < 0.3 g/day) or a partial remission (proteinuria between 0.3 and 3.5 g/day with a reduction of at least 50% from baseline) during the first year of follow-up were compared across complement groups using Kaplan–Meier survival method and log-rank test. In the ANCA-GN cohort, the association between serum complement levels and Berden histological classes was assessed using Kruskal–Wallis test and *post hoc* pairwise Wilcoxon rank sum tests in case of statistically significant results; data were graphically represented with boxplots. Multi-variable linear regression analyses for C3 and C4 were also performed, adjusting for relevant clinico-histological covariates. *β* coefficients with their 95% Confidence Intervals (CI) and *P* values were reported for each covariate. Associations between complement groups and clinical outcomes (KF and all-cause mortality) were evaluated using Kaplan–Meier survival curves and log rank test; a multi-variable Cox regression model was also fitted to explore the association between serum C3 and the risk of progression to KF, adjusting for possible confounders. Statistical analyses were performed using R version 4.5.0 and the packages stats, dplyr, tableone, survival, lm, survminer, corrplot, and ggplot2.

## RESULTS

### Membranous nephropathy cohort

A total of 44 patients with primary MN met the inclusion criteria; the main clinical features and laboratory parameters at time of diagnostic kidney biopsy are summarized in Table 1. Overall, 77% of the cohort were male and 97.7% of Caucasian ancestry. Mean age at diagnosis was 56 ± 13 years, median eGFR was 87.0 (65.7–95.7) ml/min/173 m^2^, while proteinuria and serum albumin were 4.5 (2.2–6.4) g/day and 2.5 ± 0.7 g/dl, respectively. Serum anti-PLA2R antibodies tested positive in 30/33 (90.9%) patients at diagnosis or at some point during follow-up. A total of 35 out of 44 patients received immunosuppressive therapy during follow-up (further details reported in Table 1).

**Table 1:** Baseline clinical and laboratory characteristics of 44 patients with primary MN, stratified by serum complement levels at time of diagnostic kidney biopsy.

Characteristics	Overall *N* = 44	Missing	C3-high *n* = 22	C3-low *n* = 22	*P*	C4-high *n* = 22	C4-low *n* = 22	*P*
Demographics								
Age (years)	56 ± 13	–	55 ± 12	58 ± 14	.430	60 ± 9	54 ± 15	.169
Male sex	34 (77.3%)	–	17 (77.3%)	17 (77.3%)	1	17 (77.3%)	17 (77.3%)	1
Baseline laboratory parameters								
eGFR (ml/min)	87.0 (65.7–95.7)	–	89.0 (74.0–97.2)	87.0 (65.0–93.5)	.690	82.5 (65.0–93.5)	90.5 (71.5–101.0)	.250
Serum albumin (g/dl)	2.5 ± 0.7	–	2.5 ± 0.7	2.6 ± 0.7	.508	2.4 ± 0.7	2.7 ± 0.7	.116
Proteinuria (g/day)	4.5 (2.2–6.4)	–	5.4 (2.7–7.4)	3.9 (2.0–5.4)	.227	5.5 (3.9–7.7)	2.8 (1.9–5.3)	.015
Microhematuria (yes)	32 (74.4%)	1 (2.2%)	18 (81.8%)	14 (66.7%)	.430	17 (77.3%)	15 (71.4%)	.929
Serum anti-PLA2R ab (Pos)	30 (90.9%)	11 (25%)	16 (94.1%)	14 (87.5%)	.601	16 (94.1%)	14 (87.5%)	.601
Serum C3 (mg/dl)	125 (106–134)	–	134 (131–142)	105 (95–117)	<.001	130 (120–135)	115 (95– 130)	.051
Serum C4 (mg/dl)	26 (22– 31)	–	30 (26–36)	23 (20–30)	.003	32 (29– 38)	22 (19–23)	<.001
First-line immunosuppressive treatment (*N* = 5 patients)								
Cyclic scheme RTX CNI ACTH MMF	16 (45.7%) 9 (25.7%) 5 (14.3%) 4 (11.4%) 1 (2.9%)	– – – – –	8 (47.1%) 4 (23.5%) 3 (17.6%) 2 (11.8%) 0 (0%)	8 (44.4%) 5 (27.8%) 2 (11.1%) 2 (11.1%) 1 (5.6%)	.864	10 (55.6%) 4 (22.2%) 2 (11.1%) 2 (11.1%) 0 (0%)	6 (35.3%) 5 (29.4%) 3 (17.6%) 2 (11.8%) 1 (5.9%)	.684

Results are reported as mean ± SD, median (IQR), or *N* (%), as appropriate.

ACTH, adrenocorticotropic hormone; anti-PLA2R ab, anti-phospholipase A2 receptor antibodies; CNI, calcineurin inhibitors; Cyclic scheme, iv cyclophosphamide + corticosteroids; eGFR, estimated glomerular filtration rate (calculated using the 2009 CKD-EPI formula); M, male gender; MMF, mycophenolate mofetil; RTX, rituximab.

### Association of complement levels with clinical features

C3 levels were below the lower reference limit (90 mg/dl) in five (11.4%) patients, while C4 levels were above lower reference limit (10 mg/dl) in all cases. Overall, the median C3 and C4 levels were, respectively, 125 (106–134) and 26 (22–31) mg/dl. Distribution of patients across the four complement groups is reported in Table S2A. Median serum C3 levels were 134 (131–142) mg/dl in the C3-high group and 105.0 (95–117) mg/dl in the C3-low group, while median C4 levels were 32 (29–38) mg/dl in the C4-high group and 22 (19–23) mg/dl in the C4-low group. Baseline demographics were comparable across complement groups (Table 1). There were no associations between serum C3 or C4 levels and baseline eGFR, serum albumin, or the presence of hematuria. Interestingly, proteinuria was higher in the C4-high group compared to the C4-low group (5.5 [3.9–7.7] vs. 2.8 [1.9–5.3] g/day, *p* = .015), with no differences observed across the C3-based subgroups.

### Association of complement levels with kidney histology

The main histological features of the 44 MN patients, stratified in the four complement groups, are summarized in Table 2. Notably, the degree of global GS was higher in the C3-low (8.3% [0%–25%]) compared to the C3-high subgroup (0% [0%–5%], *p* = .015). This association persisted when considering the combined percentage of global and segmental GS (C3-low: 9.5% [0%–25%]; C3-high: 0% [0%–9.2%], *p* = .032). No differences in histological features were observed in the C4 groups. Correlation analysis between complement levels and histopathological features (Fig. 1A) confirmed the inverse association between C3 levels and GS (*ρ* = −0.42; *p* = .016 for global GS; *ρ* = −0.40; *p* = .022 for global/segmental GS) and highlighted also an inverse association between C4 levels and IgG glomerular staining (*ρ* = −0.35; *p* = .028). To further explore these associations while accounting for potential confounders, we performed multi-variable linear regression analyses using serum C3 (Table S3) and C4 (Table S4) levels as dependent variables. The percentage of global GS remained independently associated with lower C3 levels (*β* = −0.68 mg/dl per 1% increase, 95% CI −1.25 to −0.10, *P* = .028). No further associations between complement levels and histological parameters emerged.

**Figure 1:**
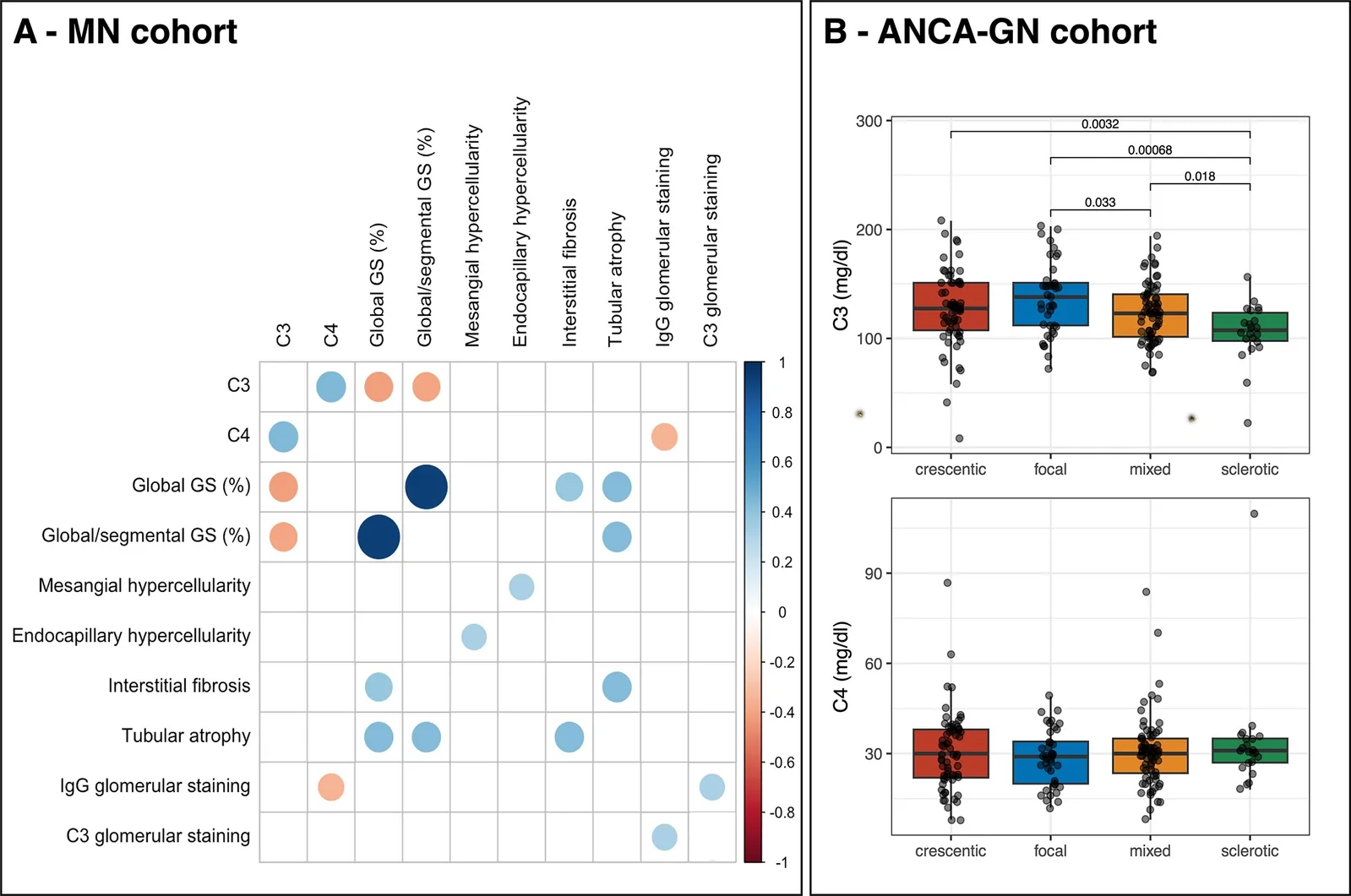
Association between key histological features and complement levels in two independent cohorts of patients with MN (A) and ANCA-GN (B). (A) Correlogram showing Spearman’s rank correlations (*ρ*) between serum complement levels and key histological features in the MN cohort. Correlation coefficients range from −1 to +1. Only statistically significant correlations (*P* < .05) are shown, with color indicating the direction of the association and color intensity-circle size reflecting the strength of the correlation. (B) Serum C3 and C4 levels in patients with ANCA-GN, stratified according to Berden classification. In case of Kruskal–Wallis *p* < .05, *P*-values for pairwise comparisons (*post hoc* pairwise Wilcoxon rank sum tests) were reported.

**Table 2:** Key histological features of 44 patients with primary MN, stratified by complement levels at time of diagnostic kidney biopsy.

Characteristics	Overall *N* = 44	Missing	C3-high *n* = 22	C3-low *n* = 22	*P*	C4-high *n* = 22	C4-low *n* = 22	*P*
Light microscopy								
Global GS^a^	1.5% (0.0–15.6)	2 (4.5%)	0.0% (0.0–5.0)	8.3% (0.0–25.0)	.015	0.0% (0.0–9.2)	4.0% (0.0–25.0)	.306
Global/segmental GS^a^	4.8% (0.0–20.0)	7 (15.9%)	0.0% (0.0–9.2)	9.5% (0.0–25.0)	.032	2.4% (0.0–15.6)	5.0% (0.0–25.0)	.423
Mesangial hypercellularity	5 (11.6%)	1 (2.3%)	3 (13.6%)	2 (9.5%)	1	4 (18.2%)	1 (4.8%)	.370
Endocapillary hypercellularity	5 (11.6%)	1 (2.3%)	1 (4.5%)	4 (19.0%)	.314	2 (9.1%)	3 (14.3%)	.956
Interstitial fibrosis Absent Mild Moderate	36 (83.7%) 6 (13.9%) 1 (2.3%)	1 (2.3%)	20 (90.9%) 2 (9.1%) 0 (0%)	16 (76.2%) 4 (19%) 1 (4.8%)	.289	17 (77.3%) 4 (18.2%) 1 (4.5%)	19 (90.5%) 2 (9.5%) 0 (0%)	.416
Tubular atrophy Absent Mild Moderate	21 (50%) 19 (45.2%) 2 (4.8%)	2 (4.5%)	10 (45.5%) 12 (54.5%) 0 (0%)	11 (55%) 7 (35%) 2 (10%)	.194	8 (38.1%) 11 (52.4%) 2 (9.5%)	13 (61.9%) 8 (38.1%) 0 (0%)	.160
Indirect immunofluorescence glomerular staining^b^								
IgG	4 (4–4)	3 (6.8%)	4 (4–4)	4 (4–4)	.448	4 (4–4)	4 (4–4)	.083
C3	3 (2–4)	4 (9.1%)	3 (2–4)	3 (2–4)	.998	3 (2–4)	3 (2–4)	.988
C1q	1 (0–1)	4 (9.1%)	0.5 (0–1)	1 (0–2)	.168	1 (0–1)	1 (0–2)	.763

Results are reported as median (IQR) or *N* (%), as appropriate.

^a^Global and/or segmental glomerulosclerosis (GS) is expressed as the percentage of affected glomeruli. ^b^Indirect immunofluorescence glomerular stainings were semi-quantitatively graded as 0–4+.

### Association of complement levels with clinical outcomes

Median follow-up time was 86 (IQR 44–126) months. Specifically, in the C3 complement groups, the cumulative probability of complete or partial remission was 11.1% at 3 months, 38.9% at 6 months, and 77.8% at 12 months in the C3-low group, and 26.3%, 36.8%, and 73.7%, respectively, in the C3-high group (log-rank test *p* = .83). Similarly, in the C4 groups, the cumulative probabilities of complete or partial remission at 3, 6, and 12 months were 22.2%, 33.3%, and 66.7% in the C4-low group, and 15.8%, 42.1%, and 84.2% in the C4-high group (log-rank test *p* = .62), respectively. No patients in the MN cohort progressed to KF or died during study follow-up, precluding analysis of the association between baseline complement levels and long-term outcomes.

### ANCA-glomerulonephritis cohort

To further explore the possible link between baseline serum complement levels and clinical/histological features, we analyzed an independent cohort of biopsy-proven ANCA-GN, grouped according to Berden histopathological classes. This cohort was part of a separate data collection and analysis initially comprising 304 patients presenting with ANCA-GN [21], with data on C3 and C4 levels at the time of kidney biopsy available for a subset of 198 (Table 3; distribution of patients across complement groups in Table S2B). This subset included 109 males (55%) and the mean age was 60 ± 14 years. Microscopic polyangiitis was the most frequent phenotype (60.9%) and there was a slight predominance of myeloperoxidase (MPO)-ANCA serotype (54.5%). Median eGFR was 20.0 (IQR 11–33.8) ml/min/1.73 m^2^ and 19.2% of patients were on kidney replacement therapy at baseline. The most common extrarenal localizations were pulmonary (44.9%), ENT (21.2%), musculoskeletal (15.7%), and cutaneous (15.2%). Overall, Berden classes were distributed as follows: 41 (20.7%) focal, 64 (32.3%) crescentic, 71 (35.9%) mixed, and 22 (11.1%) sclerotic. Induction treatment consisted of glucocorticoids (oral and/or intravenous) in all patients, in association with cyclophosphamide, rituximab, or the combination of the two in 55.1%, 15.7%, and 29.3% of cases, respectively (details in Table 3).

**Table 3:** Baseline clinical characteristics of 198 AAV patients with renal involvement, stratified by complement levels at time of diagnostic kidney biopsy.

Characteristics	Overall *N* = 198	Missing	C3-high *n* = 102	C3-low *n* = 96	*P*	C4-high *n* = 103	C4-low *n* = 95	*P*
Demographics								
Age (years)	60 ± 14	–	58 ± 14	63 ± 13	.022	59 ± 14	63 ± 13	.043
Male sex	109 (55.1%)	–	60 (58.8%)	49 (51.0%)	.338	52 (50.5%)	57 (60.0%)	.230
AAV phenotype								
GPA	75 (39.1%)	6 (3.0%)	43 (43.0%)	32 (34.8%)	.309	41 (41.4%)	34 (36.6%)	.588
MPA	117 (60.9%)		57 (57.0%)	60 (65.2%)		58 (58.6%)	59 (63.4%)	
PR3-ANCA	82 (41.4%)	–	46 (45.1%)	36 (37.5%)	.347	43 (41.7%)	39 (41.1%)	1
MPO-ANCA	108 (54.5%)	–	51 (50.0%)	57 (59.4%)	.237	52 (50.5%)	56 (58.9%)	.293
Negative ANCA	13 (6.6%)	–	5 (4.9%)	8 (8.3%)	.492	11 (10.7%)	2 (2.1%)	.032
Baseline organ involvement								
Need for KRT at onset	38 (19.2%)	–	12 (11.8%)	26 (27.1%)	.011	23 (22.3%)	15 (15.8%)	.324
Lung	89 (44.9%)	–	45 (44.1%)	44 (45.8%)	.921	47 (45.6%)	42 (44.2%)	.954
ENT	42 (21.2%)	–	27 (26.5%)	15 (15.6%)	.091	22 (21.4%)	20 (21.1%)	1
Muscoloskeletal	31 (15.7%)	–	22 (21.6%)	9 (9.4%)	.03	15 (14.6%)	16 (16.8%)	.806
Cutaneous	30 (15.2%)	–	20 (19.6%)	10 (10.4%)	.109	15 (14.6%)	15 (15.8%)	.966
Peripheral nervous system	18 (9.1%)	–	8 (7.8%)	10 (10.4%)	.702	8 (7.8%)	10 (10.5%)	.669
Ocular	6 (3.0%)	–	4 (3.9%)	2 (2.1%)	.734	4 (3.9%)	2 (2.1%)	.753
Abdominal/GI	5 (2.5%)	–	4 (3.9%)	1 (1.0%)	.402	2 (1.9%)	3 (3.2%)	.927
Central nervous system	4 (2.0%)	–	3 (2.9%)	1 (1.0%)	.657	2 (1.9%)	2 (2.1%)	1
Cardiovascular	1 (0.5%)	–	1 (1.0%)	0 (0%)	1	1 (1.0%)	0 (0%)	1
BVAS v3.0	17.0 (14.0–21.0)	5 (2.5%)	18 (15 –21)	16 (13–20)	.012	18 (15–21)	17 (14–20)	.156
Baseline laboratory parameters								
eGFR (ml/min)	20.0 (11.0–33.8)	–	23.0 (13.0–41.8)	18.0 (5.0–27.2)	.005	19.0 (9.0–26.5)	22.0 (11.5–39.0)	.084
Proteinuria (g/day)	1.6 (0.8–2.8)	23 (11.6%)	1.5 (0.7- 2.7)	1.7 (0.8–2.9)	.526	1.7 (0.9– 3.1)	1.3 (0.6–2.4)	.057
Serum C3 (mg/dl)	125 (104–148)	–	147 (131–161)	104 (93–113)	<.001	131 (115–152)	113 (96–131)	<.001
Serum C4 (mg/dl)	30 (23–36)	–	32 (26–38)	27 (20–33)	<.001	36 (32–40)	22 (17–26)	<.001
First-line induction treatment								
Iv steroids Oral steroids	128 (66.0%) 194 (99.5%)	4 (2.0%) 3 (1.5%)	58 (58.0%) 99 (99.0%)	70 (74.5%) 95 (100%)	.023 1	66 (66.0%) 100 (100%)	62 (66.0%) 94 (98.9%)	1 .979
CyC RTX + CyC RTX	109 (55.1%) 58 (29.3%) 31 (15.7%)	– – –	43 (42.2%) 43 (42.2%) 16 (15.7%)	66 (68.8%) 15 (15.6%) 15 (15.6%)	<.001 <.001 1	66 (64.1%) 27 (26.2%) 10 (9.7%)	43 (45.3%) 31 (32.6%) 21 (22.1%)	.012 .404 .028
PLEX	50 (30.5%)	34 (17.2%)	19 (23.2%)	31 (37.8%)	.062	36 (39.6%)	14 (19.2%)	.008

Results are reported as mean ± SD, median (IQR), or *N* (%), as appropriate.

CyC, cyclophosphamide; eGFR, estimated glomerular filtration rate (calculated using the 2009 CKD-EPI formula); ENT, ear nose and throat; GI, gastrointestinal; GPA, granulomatosis with polyangiitis; iv, intravenous; KRT, kidney replacement therapy (dialysis); M, male gender; MPA, microscopic polyangiitis; MPO, myeloperoxidase; PLEX, plasma exchange; PR3, proteinase-3; RTX, rituximab.

### Association of complement levels with clinical features

Serum C3 and C4 were below the lower reference cut-off in 17 (8.6%) and 3 (1.5%) patients, respectively. Baseline median serum C3 levels were 125 (104–148) mg/dl in the overall cohort, 147 (131–161) mg/dl in the C3-high group, and 104 (93–113) mg/dl in the C3-low group, while C4 levels were 30 (23–36) mg/dl in the overall cohort, 32 (26–38) and 27 (20–33) mg/dl in the C4-high and C4-low groups, respectively. Patients in the C3-low group were marginally older at diagnosis (mean age 63 ± 13 vs 58 ± 14 years old in C3-high, *p* = .022), with a similar pattern across C4 groups (mean age C4-low: 63 ± 13 years, C4-high: 59 ± 14 years; *p* = .043). Clinical AAV-phenotypes, as well as the distribution of proteinase-3- and MPO-ANCA positivity, did not differ across complement groups, while there was a lower proportion of ANCA-negative patients in the C4-low, compared to C4-high group (2.1% vs 10.7%, *p* = .032, Table 3). As for kidney presentation, the C3-low group had lower baseline eGFR (18.0 [5.0–28] vs 23.0 [13–42] ml/min per 1.73 m^2^, *p* = .005) and greater need of kidney replacement therapy at disease onset (27.1% vs 11.8%, *p* = .011) than the C3-high group. Patients in the C3-low group also showed lower frequencies of musculoskeletal symptoms (9.4% vs 21.6%, *p* = .03), and overall lower disease activity (median BVAS 16 [13–20] vs 18 [15–21], *p* = .012) than their C3-high counterpart. No clear differences in clinical presentation emerged across C4 groups.

### Association of complement levels with histological features

The distribution of Berden classes stratified by complement groups of the AAV cohort are summarized in Table 4. The sclerotic class was over-represented in the C3-low (16.7%), compared to the C3-high group (5.9%, *p* = .029). No other associations between complement groups and Berden classes were observed.

**Table 4:** Distribution of Berden classes in 198 ANCA-GN patients, stratified by complement levels at the time of diagnostic kidney biopsy.

Berden class	Overall *N* = 198	C3-high *n* = 102	C3-low *n* = 96	*P*	C4-high *n* = 103	C4-low *n* = 95	*P*
Focal	41 (20.7%)	27 (26.5%)	14 (14.6%)	.059	18 (17.5%)	23 (24.2%)	.321
Crescentic	64 (32.3%)	37 (36.3%)	27 (28.1%)	.283	33 (32.0%)	31 (32.6%)	1
Mixed	71 (35.9%)	32 (31.4%)	39 (40.6%)	.227	38 (36.9%)	33 (34.7%)	.867
Sclerotic	22 (11.1%)	6 (5.9%)	16 (16.7%)	.029	14 (13.6%)	8 (8.4%)	.352

Results are reported as *N* (%). *P*-values refer to Fisher’s exact test.

A similar pattern emerged when comparing serum complement levels across Berden classes (Fig. 1B; full data reported in Table S5). Median C3 levels differed across groups (Kruskal–Wallis *p* = .003), while C4 levels were comparable across Berden classes (Kruskal–Wallis *p* = .840). Specifically, median C3 levels were lower in the sclerotic class (107 [98–123] mg/dl), compared to those classified as focal (138 [112–151] mg/dl, *p* < .001), crescentic (127 [107–151] mg/dl, *p* = .003), or mixed (123 [101–140] mg/dl, *p* = .018). C3 levels were lower also in the mixed class, compared to the focal one (*p* = .033). In the C3 multi-variable linear regression model (Table S6), the sclerotic Berden class remained independently associated with lower serum C3 levels compared with the focal class (*β* = −19.26 mg/dl, 95% CI −37.33 to −1.19, *P* = .038). Baseline eGFR was also positively associated with C3 levels (*β* = 0.33 mg/dl per ml/min increase, 95% CI 0.10–0.55, *p* = .006). Consistently, no association was found between Berden classes and C4 levels (Table S7).

### Association between complement levels and clinical outcomes

After a median follow-up of 48 (IQR 18–53) months, 9 patients (4.5%) died and 41 (20.7%) progressed to KF. All-cause mortality did not show an association with baseline complement levels (Fig. 2). In contrast, KF was more frequent in the C3-low group compared to the C3-high one (log-rank test *p* = .011), with 1-year kidney survival rates of 83.3% vs 93.9% and 5-year survival rates of 68.5% vs 87.2%, respectively (Fig. 2A). C4 levels were not associated with the risk of KF (Fig. 2B).

**Figure 2:**
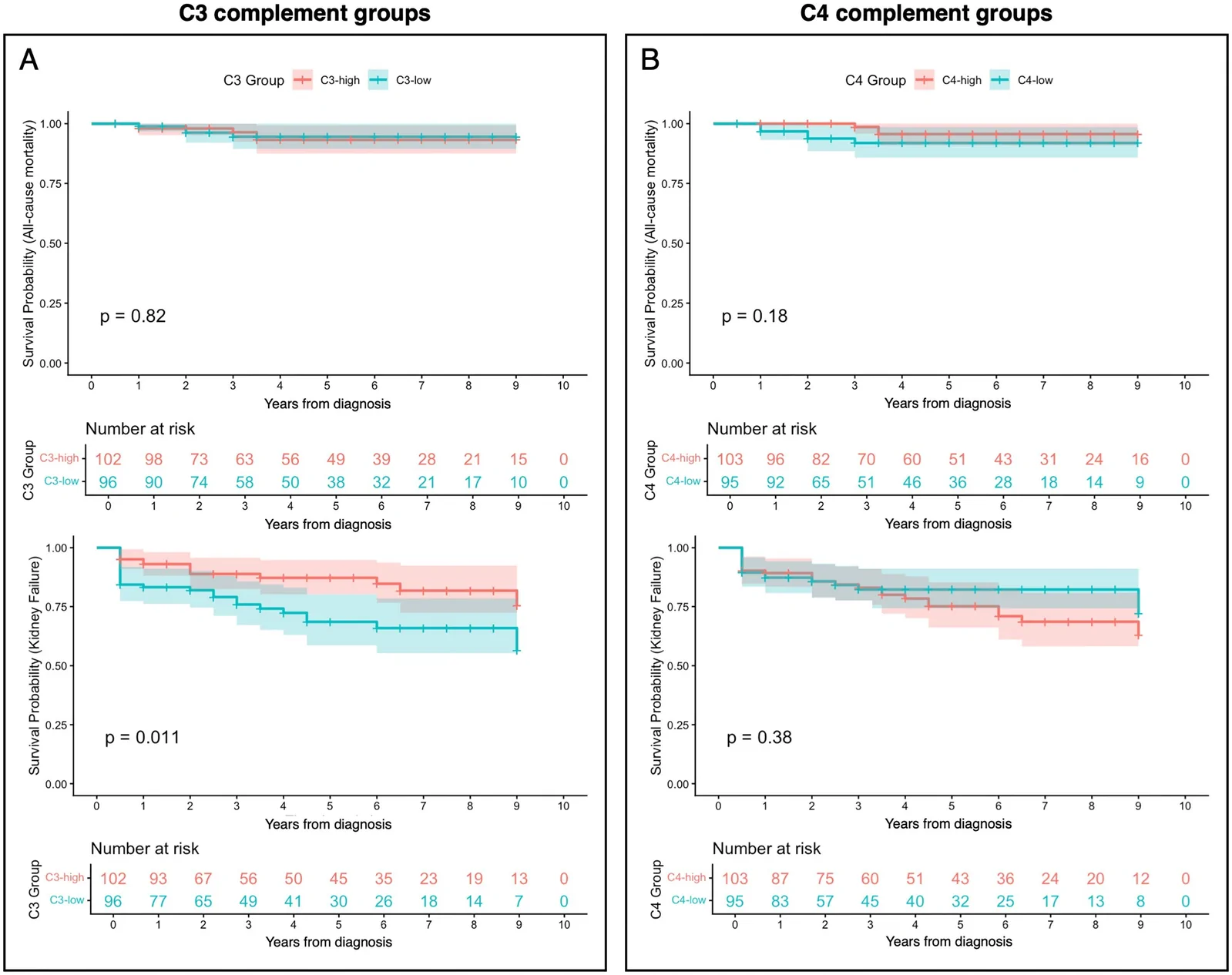
Survival curves for the risk of KF and all-cause mortality in 198 patients with ANCA-GN, stratified by C3 (panel A) and C4 (panel B) complement groups.

Decreased serum C3 remained independently associated with an increased risk of KF also in multi-variable Cox regression (HR for 10 mg/dl increase in C3: 0.91, 95% CI 0.83–1.00, *p* = .049) after adjustment for baseline eGFR, age, Berden class, and induction immunosuppression (Fig. 3).

**Figure 3:**
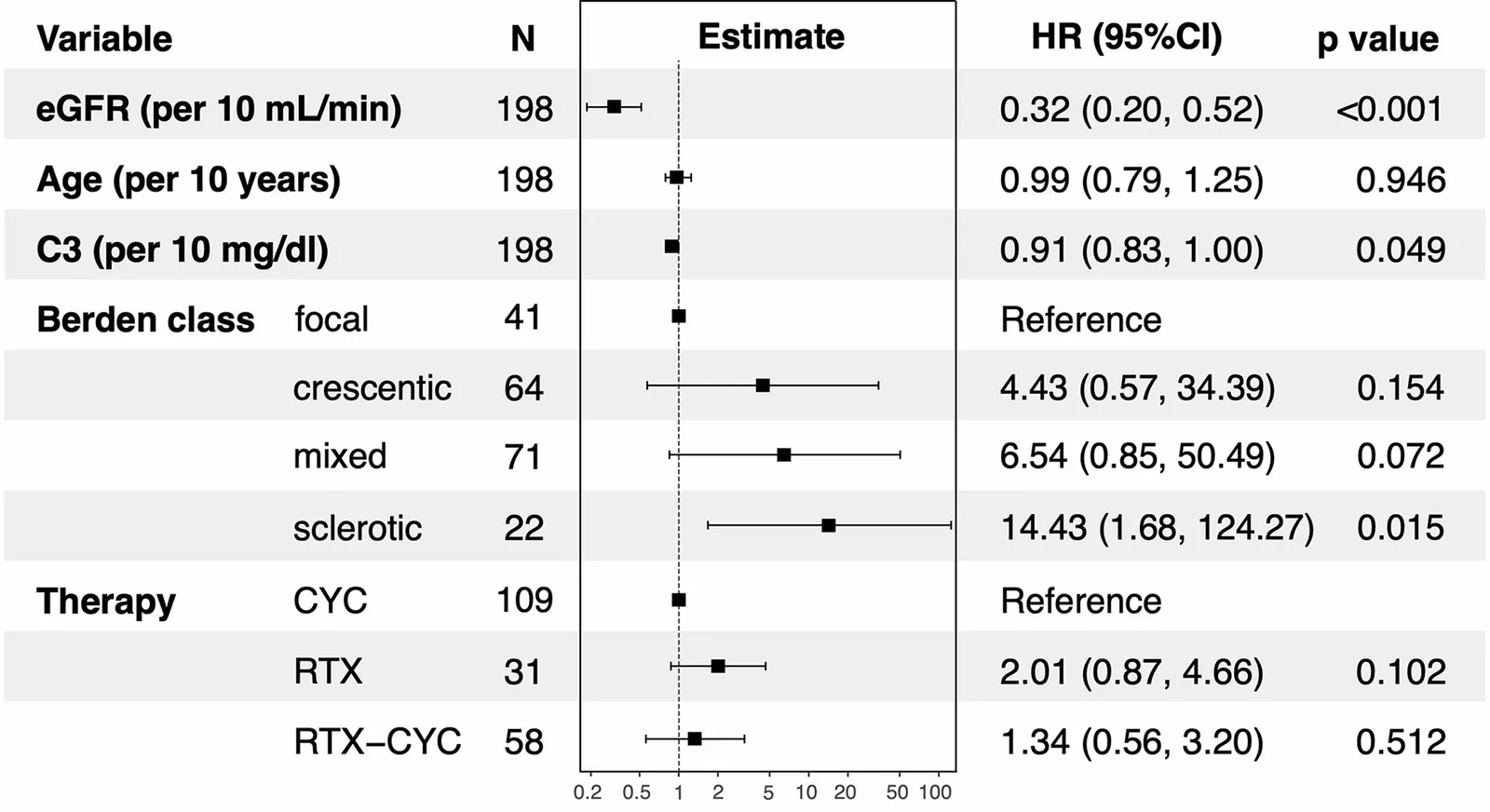
Multi-variable Cox regression analysis for the risk of KF in 198 ANCA-GN patients. HR with their 95% CI and *P* values are reported and graphically represented in the Forest plot (point refers to estimate and line represents 95% CI). CYC, cyclophosphamide; eGFR, estimated glomerular filtration rate (calculated using the 2009 CKD-EPI formula); RTX, rituximab.

## DISCUSSION

This retrospective study explored associations between widely available biomarkers of the complement system, namely, serum C3 and C4 levels, and clinic-pathological features in MN and ANCA-GN.

First, we observed an association between lower C3 levels and glomerular sclerosis, which emerged both in MN and ANCA-GN. While we are not aware of other studies specifically addressing this question in MN, our results are consistent with previous observations in ANCA-GN, where similar results were reported as secondary findings in studies designed to investigate different objectives. For instance, Manenti *et al*. found that AAV patients with reduced serum C3 levels, compared to the group with normal C3, had a higher proportion of Berden sclerotic class [25]. However, it must be stressed that lower C3 has been associated also with other pathological lesions in ANCA-GN, such as crescentic lesions [26] and features of thrombotic microangiopathy [25]. Interestingly, the association between C3 levels in the low/low–normal range and histological features of chronic damage has been reported in different contexts as well, such as anti-Glomerular Basement Membrane (GBM) disease [27, 28], lupus nephritis [29], and diabetic nephropathy [30]. These findings highlight a potential cross-disease role of the complement system in chronic kidney injury. However, the relevance of this association to pathophysiology remains unclear.

A first possibility is that C3 hypocomplementemia reflects complement activation, with subsequently increased consumption of the C3 component, suggesting a role of complement activation in driving sclerotic lesions. This hypothesis is in line with different pieces of evidence, ranging from basic science to translational studies. For instance, complement anaphylatoxins promote epithelial to mesenchymal transition [31] as well as local recruitment and activation of macrophages, which in turn produce pro-fibrotic cytokines, driving extracellular matrix remodeling and collagen deposition [31, 32]. The pathogenetic role of complement in driving kidney fibrosis is also supported by animal models. For example, in a mouse model of focal segmental GS induced by Adriamycin, genetic ablation of C3 prevents the development of GS and tubulointerstitial injury [33]. As for translational human studies, urine proteomic signatures of complement activation have been associated with more severe glomerular and interstitial sclerosis in diabetic nephropathy [34]. Furthermore, increased levels of urinary complement activation biomarkers (specifically C5b9 and C5a) have been detected in focal segmental GS, but not minimal change disease [35].

In this setting, what drives complement activation remains unknown. Genetic polymorphisms in alternative pathway components may play a role in contributing to excessive complement activation. As recently shown in the context of Immunoglobulin A (IgA) nephropathy [36], genetic variants may disrupt the balance between complement regulators and activators, enhancing complement activation and C3 consumption, which is then sustained by intra-renal pathological processes. Furthermore, CKD itself has been linked to hyperreactivity of the alternative complement pathway, as reflected by higher levels of circulating complement activators and active fractions. The degree of this activation has been proposed to increase with declining kidney function, and its role as contributor to renal injury and CKD-related complication remains an active area of investigation [37].

In addition to complement activation, serum C3 levels may also be affected by rates of C3 generation. First, C3 is a positive acute-phase reactant produced by the liver and its synthesis increases during systemic inflammation [38]. This mechanism may be driving the positive association that we observed between vasculitis activity, as captured by the BVAS score, and C3 levels. Moreover, the kidney itself can synthetize complement components, including C3, with expression documented across a variety of cell types, both in glomeruli and in the tubulointerstitial compartment [39]. While intra-renal expression of complement components only marginally contributes to circulating levels of complement factors under normal circumstances, it can increase during kidney inflammation, as documented in the setting of acute allograft rejection [40]. The association between lower serum C3 and chronic kidney lesions may therefore reflect reduced systemic and/or intra-renal synthesis of C3, compared to cases with more pronounced immunological activation, either at the local or systemic level.

Furthermore, we cannot rule out that lower levels of circulating C3 may represent a consequence of glomerular scarring rather than a causal factor. In fact, podocyte injury decreases the tension on the GBM, thereby increasing its permeability [41] and facilitating urinary loss of proteins, including complement components. However, this does not seem to be a quantitatively relevant mechanism in these study cohorts, given the lack of association between proteinuria and serum C3/C4 levels.

Importantly, lower baseline C3 was also associated with more severe kidney involvement and poorer kidney prognosis in ANCA-GN. This association remained evident also after adjusting for several possible confounders, including Berden class and eGFR at presentation. The association between lower serum C3 and worse kidney outcomes has been consistently replicated in several other ANCA-GN cohorts [26, 42–47], as well as in other disease contexts, such as focal segmental GS [48], IgA nephropathy [49, 50], anti-GBM disease [27, 28], or lupus nephritis [29]. Lower serum C3 levels have been independently associated with poorer kidney prognosis in a cohort of 99 patients with idiopathic MN as well [51]. Unfortunately, we could not assess the prognostic significance of serum C3 levels in the MN cohort, due to the lack of kidney events during the relatively short follow-up.

In contrast to serum C3, serum C4 levels did not predict kidney outcomes in patients with ANCA-GN. This is in line with other reports [52] and with the body of evidence supporting that complement activation in AAV is driven by the alternative pathway [53].

While our data are not informative regarding the prognostic impact of complement levels in MN, higher serum C4 levels have been associated with worse kidney prognosis in a Chinese MN cohort [54], as well as in IgA nephropathy [49]. The mechanisms underlying these findings remain unclear. In our MN cohort, higher serum C4 was associated with greater proteinuria, while this was not observed in the aforementioned Chinese cohort [54]. We also noted a negative association between serum C4 levels and the intensity of IgG, but not C3, glomerular staining in MN. Further data are needed to assess the reproducibility and significance of these findings.

Several limitations of our study should be acknowledged, including its retrospective design, the lack of centralized review of kidney biopsies, the relatively small size of the MN cohort, and missing data on plasma exchange. In addition, the relatively low number of KF events in the ANCA-GN cohort limited the number of covariates that could be included in the Cox regression analysis. While we deliberately choose to focus on serum C3/C4 levels given their widespread availability in routine clinical practice, interpretating the results is difficult, given the diverse and possibly concomitant factors that can affect the levels of biomarkers. Additional information on tissue, urine, or circulating complement split products levels may shed better light on the underlying mechanisms.

In conclusion, lower circulating C3 levels correlated with greater histological chronicity in both MN and ANCA-GN. In ANCA-GN, reduced serum C3 additionally identified patients with more severe kidney disease and poorer kidney outcomes. These findings, together with a growing body of evidence, reinforce the central role of complement activation in the pathophysiology of ANCA-GN and MN, as well as other glomerular diseases. Complement activation likely contributes to diverse pathogenetic processes—ranging from active inflammation to fibrosis—across different stages of disease, adding further complexity.

Future research should aim to more precisely characterize the spectrum of complement activation in individual patients and at varying disease stages, ideally using biomarkers that more directly reflect underlying pathophysiological mechanisms than serum C3 and C4 levels. This will help guide personalized treatment strategies and optimize the use of the rapidly expanding armamentarium of complement-modulating therapeutics [55].

## Supplementary Material

sfag350_Supplemental_Files

## Data Availability

The data underlying this article will be shared on reasonable request to the corresponding author.
