# Supplementary material for: Lower serum C3 levels are associated with glomerulosclerosis in membranous nephropathy and ANCA-glomerulonephritis
Source: Clin Kidney J. 2026 Oct 5;19(11):sfag350. doi: 10.1093/ckj/sfag350 (PMC13724270; doi:10.1093/ckj/sfag350)
Supplement: Supplementary file 1 [file sfag350_supplemental_files.zip › Figure S1.pdf]

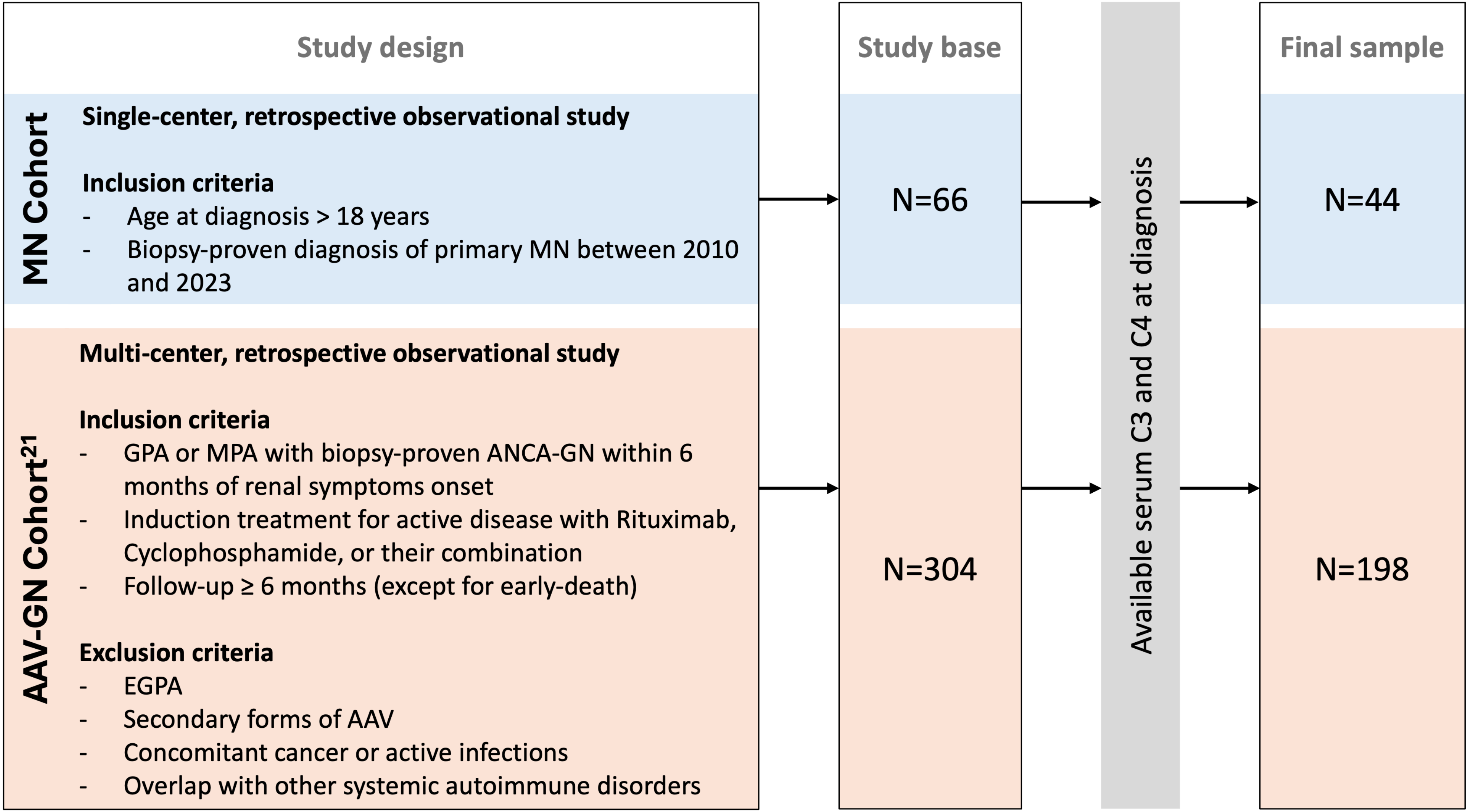
Study design
Study base
Final sample
MN Cohort
Single-center, retrospective observational study
Inclusion criteria
- Age at diagnosis > 18 years
- Biopsy-proven diagnosis of primary MN between 2010 and 2023
N=66
N=44
Available serum C3 and C4 at diagnosis
AAV-GN Cohort[21]
Multi-center, retrospective observational study
Inclusion criteria
- GPA or MPA with biopsy-proven ANCA-GN within 6 months of renal symptoms onset
- Induction treatment for active disease with Rituximab, Cyclophosphamide, or their combination
- Follow-up ≥ 6 months (except for early-death)
Exclusion criteria
- EGPA
- Secondary forms of AAV
- Concomitant cancer or active infections
- Overlap with other systemic autoimmune disorders
N=304
N=198
