# Supplementary material for: Lower serum C3 levels are associated with glomerulosclerosis in membranous nephropathy and ANCA-glomerulonephritis
Source: Clin Kidney J. 2026 Oct 5;19(11):sfag350. doi: 10.1093/ckj/sfag350 (PMC13724270; doi:10.1093/ckj/sfag350)
Supplement: Supplementary file 1 [file sfag350_supplemental_files.zip › Supplementary material_submission II.docx]

**SUPPLEMENTARY MATERIAL**

| **Table S1 –** EUVAS centers enrolling patients in the ANCA-GN cohort | Pag 2 |
| --- | --- |
| **Table S2** – Distribution of patients across complement groups in the MN (A) and ANCA-GN cohort (B) | Pag 2 |
| **Table S3** – Multivariable linear regression analysis of factors associated with C3 levels (mg/dL) in the MN cohort | Pag 3 |
| **Table S4** - Multivariable linear regression analysis of factors associated with C4 levels (mg/dL) in the MN cohort | Pag 3 |
| **Table S5** – Distribution of Berden classes according to C3 and C4 levels in 198 ANCA-GN patients, stratified by assay-specific lower reference limits | Pag 3 |
| **Table S6** – Multivariable linear regression analysis of factors associated with C3 levels (mg/dL) in the ANCA-GN cohort | Pag 4 |
| **Table S7** - Multivariable linear regression analysis of factors associated with C4 levels (mg/dL) in the ANCA-GN cohort | Pag 4 |
| **Figure S1 –** Flowchart showing the patient selection process for the MN cohort and ANCA-GN cohort | Pag 5 |

**Table S1 –** EUVAS centers enrolling patients in the ANCA-GN cohort

| **Center, city** | **Country** |
| --- | --- |
| Medical University Hospital Innsbruck, Innsbruck | Austria |
| Agram Special Hospital, Zagreb | Croatia |
| Trinity Health Kidney Centre, Dublin | Ireland |
| ASST Spedali Civili of Brescia, Brescia | Italy |
| Sant'Orsola Malpighi Hospital, Bologna | Italy |
| Careggi University Hospital, Florence | Italy |
| ASST Santi Paolo e Carlo, Milan | Italy |
| IRCCS San Gerardo dei Tintori, Monza | Italy |
| Karolinska University Hospital, Stockholm | Sweden |
| Imperial College Healthcare NHS Trust, London | United Kingdom |
| Addenbrooke's hospital, Cambridge | United Kingdom |

**Table S2** – Distribution of patients across complement groups in the MN (A) and ANCA-GN cohort (B)

A) MN cohort (n=44)

|  | **C3-low** | **C3-high** |
| --- | --- | --- |
| **C4-low** | 15 | 7 |
| **C4-high** | 7 | 15 |

B) ANCA-GN cohort (n=198)

|  | **C3-low** | **C3-high** |
| --- | --- | --- |
| **C4-low** | 59 | 36 |
| **C4-high** | 37 | 66 |

**Table S3** – Multivariable linear regression analysis of factors associated with C3 levels (mg/dL) in the MN cohort

| **Variable** |  | **β coefficient** | **95% CI** | **p value** |
| --- | --- | --- | --- | --- |
| **Age** | Per 1-year increase | −0.13 | −0.75, 0.49 | 0.680 |
| **Sex** | Female | Ref. | — | — |
|  | Male | −14.82 | −31.84, 2.20 | 0.098 |
| **eGFR** | Per mL/min increase | −0.24 | −0.61, 0.13 | 0.206 |
| **Proteinuria** | Per g/day increase | 1.05 | −0.57, 2.66 | 0.212 |
| **Global glomerulosclerosis** | Per 1% increase | −0.68 | −1.25, −0.10 | 0.028 |
| **Interstitial fibrosis*** | Per 1-unit increase | −1.07 | −16.01, 13.88 | 0.890 |
| **IgG capillary staining**° | Per 1-unit increase | −1.43 | −16.00, 13.14 | 0.849 |

°indirect immunofluorescence glomerular stainings were semi-quantitatively graded as 0-4+. *Interstitial fibrosis was semi-quantitatively graded as absent-mild-moderate-severe; eGFR = estimated glomerular filtration rate

**Table S4** - Multivariable linear regression analysis of factors associated with C4 levels (mg/dL) in the MN cohort

| **Variable** |  | **β coefficient** | **95% CI** | **p value** |
| --- | --- | --- | --- | --- |
| **Age** | Per 1-year increase | −0.01 | −0.24, 0.22 | 0.940 |
| **Sex** | Female | Ref. | — | — |
|  | Male | −0.55 | −6.86, 5.76 | 0.866 |
| **eGFR** | Per mL/min increase | −0.10 | −0.23, 0.04 | 0.172 |
| **Proteinuria** | Per g/day increase | 0.42 | −0.18, 1.02 | 0.178 |
| **Global glomerulosclerosis** | Per 1% increase | −0.19 | −0.41, 0.02 | 0.088 |
| **Interstitial fibrosis*** | Per 1-unit increase | 4.19 | −1.35, 9.74 | 0.148 |
| **IgG capillary staining**° | Per 1-unit increase | −5.38 | −10.78, 0.03 | 0.060 |

°indirect immunofluorescence glomerular stainings were semi-quantitatively graded as 0-4+. *Interstitial fibrosis was semi-quantitatively graded as absent-mild-moderate-severe; eGFR = estimated glomerular filtration rate

**Table S5** – Serum C3 and C4 levels across Berden classes in 198 ANCA-GN patients

|  | **Focal**  **(n=41)** | **Crescentic**  **(n=64)** | **Mixed**  **(n=71)** | **Sclerotic**  **(n=22)** | **p** |
| --- | --- | --- | --- | --- | --- |
| **C3 (mg/dL)** | 138  (112 – 151) | 127  (107 – 151) | 123  101 – 140 | 107  98 – 123 | 0.003 |
| **C4 (mg/dL)** | 29  (20 – 34.0) | 30  (22.0 – 38.0) | 30  23 – 35 | 31  27 – 35 | 0.84 |

Results are reported as median and IQR.

**Table S6** – Multivariable linear regression analysis of factors associated with C3 levels (mg/dL) in the ANCA-GN cohort

| **Variable** |  | **β coefficient** | **95% CI** | **p value** |
| --- | --- | --- | --- | --- |
| **Age** | Per 1-year increase | −0.11 | −0.44, 0.23 | 0.525 |
| **Sex** | Female | Ref. | — | — |
|  | Male | 3.32 | −5.67, 12.30 | 0.470 |
| **eGFR** | Per mL/min increase | 0.33 | 0.10, 0.55 | 0.006 |
| **ANCA specificity** | MPO | Ref. | — | — |
|  | PR3 | 5.99 | −4.32, 16.30 | 0.256 |
|  | MPO-PR3 double + | −20.09 | −47.65, 7.46 | 0.155 |
|  | Negative | −2.88 | −20.79, 15.04 | 0.753 |
| **BVAS score** | Per 1-point increase | 0.85 | −0.02, 1.72 | 0.057 |
| **Organ involvement** | Lung | −0.35 | −9.72, 9.03 | 0.942 |
|  | ENT | 0.57 | −11.96, 13.11 | 0.928 |
|  | Musculoskeletal | 2.98 | −10.32, 16.29 | 0.661 |
| **Berden class** | Focal | Ref. | — | — |
|  | Crescentic | −2.12 | −16.24, 12.00 | 0.769 |
|  | Mixed | −3.83 | −17.36, 9.71 | 0.580 |
|  | Sclerotic | −19.26 | −37.33, −1.19 | 0.038 |

BVAS = Birmingham Vasculitis Activity Score v3.0; eGFR = estimated glomerular filtration rate; ENT = ear nose and throat; MPO = myeloperoxidase; PR3 = proteinase 3

**Table S7** - Multivariable linear regression analysis of factors associated with C4 levels (mg/dL) in the ANCA-GN cohort

| **Variable** |  | **β coefficient** | **95% CI** | **p value** |
| --- | --- | --- | --- | --- |
| **Age** | Per 1-year increase | −0.11 | −0.26, 0.03 | 0.114 |
| **Sex** | Female | Ref. | — | — |
|  | Male | −0.15 | −3.95, 3.65 | 0.938 |
| **eGFR** | Per mL/min increase | −0.08 | −0.17, 0.02 | 0.115 |
| **ANCA specificity** | MPO | Ref. | — | — |
|  | PR3 | 1.59 | −2.77, 5.96 | 0.476 |
|  | MPO-PR3 double + | 6.45 | −5.22, 18.11 | 0.280 |
|  | Negative | 4.23 | −3.36, 11.81 | 0.276 |
| **BVAS score** | Per 1-point increase | 0.08 | −0.29, 0.44 | 0.680 |
| **Organ involvement** | Lung | −1.37 | −5.34, 2.60 | 0.500 |
|  | ENT | 0.96 | −4.35, 6.27 | 0.724 |
|  | Musculoskeletal | −3.56 | −9.20, 2.07 | 0.217 |
| **Berden class** | Focal | Ref. | — | — |
|  | Crescentic | −1.68 | −7.65, 4.30 | 0.584 |
|  | Mixed | 0.13 | −5.60, 5.86 | 0.965 |
|  | Sclerotic | 2.79 | −4.86, 10.44 | 0.476 |

BVAS = Birmingham Vasculitis Activity Score v3.0; eGFR = estimated glomerular filtration rate; ENT = ear nose and throat; MPO = myeloperoxidase; PR3 = proteinase 3

**Figure S1 –** Flowchart showing the patient selection process for the MN cohort and ANCA-GN cohort

ANCA-GN = ANCA-associated vasculitis glomerulonephritis; AAV= ANCA-associated vasculitis, EGPA = eosinophilic granulomatosis with polyangiitis, GPA = granulomatosis with polyangiitis; MN = membranous nephropathy; MPA = microscopic polyangiitis.
